# Anti-Apolipoprotein A-1 IgG Predict All-Cause Mortality and Are Associated with Fc Receptor-Like 3 Polymorphisms

**DOI:** 10.3389/fimmu.2017.00437

**Published:** 2017-04-18

**Authors:** Panagiotis Antiochos, Pedro Marques-Vidal, Julien Virzi, Sabrina Pagano, Nathalie Satta, Oliver Hartley, Fabrizio Montecucco, François Mach, Zoltán Kutalik, Gerard Waeber, Peter Vollenweider, Nicolas Vuilleumier

**Affiliations:** ^1^Department of Internal Medicine, Lausanne University Hospital, Lausanne, Switzerland; ^2^Division of Laboratory Medicine, Department of Genetics and Laboratory Medicine, Geneva University Hospitals, Geneva, Switzerland; ^3^Faculty of Medicine, Department of Human Protein Sciences, University of Geneva, Geneva, Switzerland; ^4^Faculty of Medicine, Department of Pathology and Immunology, University of Geneva, Geneva, Switzerland; ^5^Division of Cardiology, Foundation for Medical Researches, Department of Medical Specialties, University of Geneva, Geneva, Switzerland; ^6^First Clinic of Internal Medicine, Department of Internal Medicine, University of Genoa, Genoa, Italy; ^7^Institute of Social and Preventive Medicine, University Hospital of Lausanne, Lausanne, Switzerland; ^8^Swiss Institute of Bioinformatics, Lausanne, Switzerland

**Keywords:** autoimmunity, autoantibodies, apolipoprotein A-1, mortality, genome-wide association study, Fc receptor-like 3

## Abstract

**Background:**

Autoantibodies against apolipoprotein A-1 (anti-apoA-1 IgG) have emerged as an independent biomarker for cardiovascular disease and mortality. However, their association with all-cause mortality in the community, as well as their genetic determinants, have not been studied.

**Objective:**

To determine whether anti-apoA-1 IgG: (a) predict all-cause mortality in the general population and (b) are associated with single-nucleotide polymorphisms (SNPs) in a genome-wide association study (GWAS).

**Methods:**

Clinical, biological, and genetic data were obtained from the population-based, prospective CoLaus study, including 5,220 participants (mean age 52.6 years, 47.3% men) followed over a median duration of 5.6 years. The primary study outcome was all-cause mortality.

**Results:**

After multivariate adjustment, anti-apoA-1 IgG positivity independently predicted all-cause mortality: hazard ratio (HR) = 1.54, 95% confidence interval (95% CI): 1.11–2.13, *P* = 0.01. A dose–effect relationship was also observed, each SD of logarithmically transformed anti-apoA-1 IgG being associated with a 15% increase in mortality risk: HR = 1.15, 95% CI: 1.02–1.28, *P* = 0.028. The GWAS yielded nine SNPs belonging to the Fc receptor-like 3 (*FCRL3*) gene, which were significantly associated with anti-apoA-1 IgG levels, with the lead SNP (rs6427397, *P* = 1.54 × 10^−9^) explaining 0.67% of anti-apoA-1 IgG level variation.

**Conclusion:**

Anti-apoA-1 IgG levels (a) independently predict all-cause mortality in the general population and (b) are linked to *FCRL3*, a susceptibility gene for numerous autoimmune diseases. Our findings indicate that preclinical autoimmunity to anti-apoA-1 IgG may represent a novel mortality risk factor.

## Introduction

Autoimmune diseases (ADs) represent a major health issue affecting up to 10% of the population (1–4). Multiple studies have established the relationship between a chronic inflammatory state due to clinical or quiescent autoimmunity and poor outcomes in various clinical settings (5, 6). Marking sustained B-cell activation, presence of autoantibodies specifically represents a signature of humoral autoimmunity, which can drive disease pathogenesis even in the absence of overt clinical AD (7–9).

More than a decade ago, early clinical studies have supported the role of autoantibodies against apolipoprotein A-1 (anti-apoA-1 IgG), the principal component of high-density lipoproteins (HDLs), as mediators of chronic low-grade inflammation and predictors of unfavorable outcomes in patients with ADs (10–17). Subsequently, *in vitro* and animal studies demonstrated the ability of these autoantibodies to elicit a pro-inflammatory and pro-atherogenic response through interaction with the TLR2/TLR4/CD14 complex, followed by NF-κB and MAPK downstream activation as the main molecular pathway (18–24).

Recently, due to these pro-inflammatory and pro-atherogenic properties, anti-apoA-1 IgG gained interest as independent biomarkers for incident cardiovascular (CV) disease (CVD) and mortality, as well as potential therapeutic targets for immune-modulating interventions, in high CV risk populations (16, 19, 20, 22, 25–28). Moreover, similar to what has been reported with other autoantibodies (7), our group recently demonstrated that elevated anti-apoA-1 IgG levels were present in up to 20% of individuals in the general population and associated with prevalent CVD independently of traditional CV risk factors (25). Nevertheless, the association of anti-apoA-1 IgG with all-cause mortality in the general population has not yet been studied.

From another point of view, while rare autoimmunity syndromes can result from monogenic mutations, common human ADs are complex disorders arising from the interaction between polygenic and environmental risk factors, disrupting mechanisms of immune tolerance. In recent years, genome-wide association studies (GWASs) have provided insight into the subtle immune dysregulation caused by common genetic variants that predispose to clinical autoimmunity (29, 30) and autoantibody production in particular (31–34).

Although the risk attributable to most of the identified individual nucleotide variants is modest, modern GWAS have the potential to provide an unbiased view of biological pathways that drive autoimmunity (1–4). However, despite the associations of anti-apoA-1 IgG with adverse outcomes in different patient populations, no study has so far investigated common genetic variants potentially related to their serum values.

Thus, the goal of the present study was twofold. We aimed to determine whether anti-apoA-1 IgG: (a) predict all-cause mortality in the general population in a prospective study and (b) are associated with single-nucleotide polymorphisms (SNPs) in a GWAS.

## Methods

### Study Population and Design

Clinical and biological data were obtained from the CoLaus study, a population-based prospective cohort of 6,733 participants recruited between 2003 and 2006 in the city of Lausanne, Switzerland. Of the initial baseline sample of 6,733 participants, 5,220 (mean age 52.6 ± 10.7 years, 47.3% men) had complete clinical and biological data over a median follow-up (FU) time of 5.6 years and were included in the prospective analysis. A detailed description of the study design, variables, and sampling procedures has been reported elsewhere (35).

All participants attended the outpatient clinic of the University Hospital of Lausanne. Clinical data and fasting venous blood samples were collected from each participant by trained field interviewers during a single visit lasting about 60 min. Blood pressure and heart rate were measured three consecutive times using an automated sphygmomanometer (Omron^®^ HEM-907, Matsusaka, Japan), and the average of the last two measurements was used. Body weight and height were measured with participants standing without shoes in light indoor clothes. Body weight was measured in kilograms to the nearest 100 g using a Seca^®^ scale, and height was measured to the nearest 5 mm using a Seca^®^ height gauge. Body mass index (BMI) was calculated as weight (kg)/height (m^2^). Hypertension was defined as a systolic blood pressure ≥140 mm Hg and/or a diastolic blood pressure ≥90 mm Hg and/or the presence of anti-hypertensive treatment. Diabetes mellitus was defined as fasting plasma glucose ≥7.0 mmol/l and/or oral or insulin anti-diabetic treatment. History of CVD was defined by the presence of myocardial infarction, angina pectoris, percutaneous revascularization or bypass grafting for ischemic heart disease, and stroke or transient ischemic attack and assessed according to standardized medical records (35). History of ADs was obtained *via* questionnaire. Estimated glomerular filtration rate (eGFR) was calculated by the simplified “Modification of Diet in Renal Disease” prediction equation. Absolute risk for CVD was computed using the Systematic Coronary Risk Evaluation algorithm (36).

Venous blood samples were drawn after an overnight fast, and assays were performed on fresh plasma samples within 2 h of blood collection for standard lipid profile and on unthawed serum aliquots for anti-apoA-1 IgG determination, (see below) that were immediately processed and stored at −80°C. Standard lipid profile was performed by the CHUV Clinical Laboratory using a Modular P apparatus (Roche Diagnostics, Switzerland). The following analytical procedures (with maximum inter- and intra-batch CVs) were used: total cholesterol by the “CHOD-PAP” method (1.6–1.7%); HDL cholesterol by the “CHOD-PAP/PEG/Cyclodextrin” method (3.6–0.9%); triglycerides by the “GPO-PAP” method (2.9–1.5%); glucose by glucose dehydrogenase (2.1–1.0%); and serum creatinine by the Jaffe kinetic compensated method (2.9–0.7%).

### Determination of Anti-apoA-1 IgG Levels

Autoantibodies against apolipoprotein A-1 were measured as previously described (19, 22, 37), using the CoLaus study (2003–2006) frozen serum aliquots, stored at −80°C. Maxisorp plates (Nunc™, Denmark) were coated with purified, human-derived delipidated apolipoprotein A-1 (20 µg/ml; 50 µl/well) for 1 h at 37°C. After being washed, all wells were blocked for 1 h with 2% bovine serum albumin (BSA) in a phosphate buffer solution (PBS) at 37°C. Participants’ samples were also added to a non-coated well to assess individual non-specific binding. After six washing cycles, a 50 µl/well of signal antibody (alkaline phosphatase-conjugated anti-human IgG; Sigma-Aldrich, St. Louis, MO, USA), diluted 1:1,000 in a PBS/BSA 2% solution, was added and incubated for 1 h at 37°C. After washing six more times, phosphatase substrate *p*-nitrophenyl phosphate disodium (Sigma-Aldrich) dissolved in a diethanolamine buffer (pH 9.8) was added and incubated for 20 min at 37°C (Molecular Devices™ Versa Max). Optical density (OD) was determined at 405 nm, and each sample was tested in duplicate. Corresponding non-specific binding was subtracted from mean OD for each sample. The specificity of detection was assessed using conventional saturation tests by Western blot analysis.

As previously described (19, 22, 37), elevated levels of anti-apoA-1 IgG were set at an OD cut-off of OD >0.64, corresponding to the 97.5th percentile of a reference population of 140 healthy blood donors. In order to limit the impact of inter-assay variation, we further calculated an index consisting in the ratio between sample net absorbance and the positive control net absorbance × 100. The index value corresponding to the 97.5th percentile of the normal distribution was 37. Accordingly, to be considered as positive (presenting elevated anti-apoA-1 IgG levels), samples had to display both an absorbance value >0.64 OD and an index value ≥37%.

### Genome-Wide Association Study

Genotyping was performed using the Affymetrix GeneChip^®^ Human Mapping 500K array set and genotypes were called using BRLMM. SNPs with a call rate <70% and individuals with call rate <90% were excluded from further analysis. Participants found to be of non-European ancestry by principal component analysis of the genotype data were also excluded, leaving 5,402 participants eligible for GWAS. Imputation was performed using IMPUTE version 0.2.0 and CEU haplotypes from HapMap release 21. The dataset used for imputation consisted in 390,631 genotyped SNPs with a call rate >0.9, Hardy–Weinberg *P*-value >10^−7^, and MAF >1%.

Before performing the GWAS, anti-apoA-1 IgG levels were adjusted for age, sex, and ancestry principal components. The residuals were then inverse normal quantile transformed and regressed onto genetic allele dosages. To fine map the genome-wide significant association at the *FCRL2/3* locus, we re-imputed the 400-kb window around the top HapMap-associated SNP using haplotypes from the HRC reference panel.

### Study Endpoints

The primary study endpoint was overall mortality, but specific causes of death were also considered. All deaths and related causes were adjudicated by an independent panel of internal medicine physicians, blinded to all study variables.

### Statistics

Statistical analyses were conducted using Stata v14.1 (Stata Corp., TX, USA) and MatLab v8.3 (MathWorks, MA, USA). Bivariate analysis of continuous variables was performed using Student’s or Mann–Whitney test as appropriate, while analysis of categorical variables was performed using chi-square test. The association of anti-apoA-1 IgG levels with all-cause mortality was assessed by the log-rank test and by Cox proportional hazards regression, adjusting for age, gender, hypertension, diabetes, smoking, BMI, eGFR, HDL and low-density lipoprotein (LDL) cholesterol, baseline CVD, and AD. Anti-apoA-1 IgG concentrations were natural log transformed to account for skewed distributions, and results were expressed as hazard ratios (HRs) and 95% confidence intervals (95% CIs). Considering a two-sided alpha of 0.05, our study had 80% power to detect a relative risk for all-cause mortality of 1.45 in participants positive for anti-apoA-1 IgG. All tests were two tailed, and *P* values <0.05 were considered as statistically significant.

## Results

**Figure 1:**
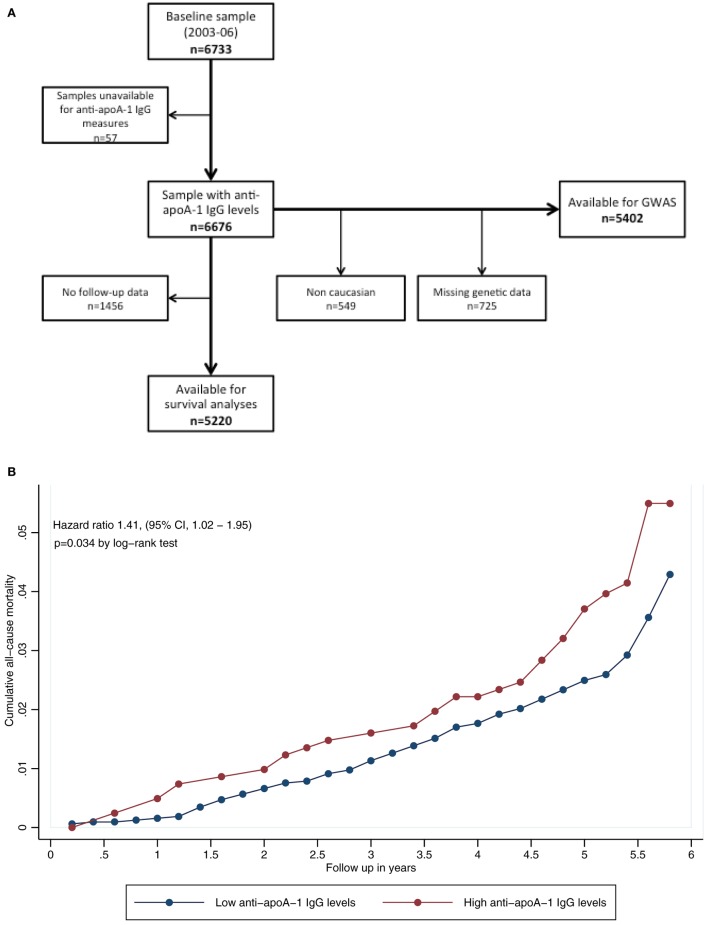
**(A)** Participants’ selection procedure. **(B)** Kaplan–Meier curves for all-cause mortality, according to autoantibodies against apolipoprotein A-1 (anti-apoA-1 IgG) status. *P*-value according to log-rank test (*P* = 0.034).

Figure 1A shows the participants’ selection procedure, and Table S1 in Supplementary Material summarizes the baseline characteristics of participants, with or without FU data. Overall, subjects lost at FU were more likely to be smokers, hypertensive, obese, and with a less favorable lipid profile than subjects included in the analysis, but did not differ with regards to anti-apoA-1 IgG positivity or serum levels.

During FU, 191 deaths (3.7%) occurred. The three major causes of death were cancer, CVD, and infectious diseases. Participants who died presented with a significantly higher prevalence of anti-apoA-1 IgG positivity (26.7 vs. 19.6%, *P* = 0.016) and higher anti-apoA-1 IgG levels (median [interquartile range]: 0.43 [0.30–0.70] vs. 0.39 [0.25–0.59] AU, *P* = 0.007), than participants alive at FU (Table 1). Of note, median anti-apoA-1 IgG values of anti-apoA-1 IgG-positive subjects who died at FU were 0.89 [0.79–1.05] AU.

**Table 1:** **Baseline characteristics of the sample according to all-cause mortality during follow-up**.

	Overall (*n* = 5,220)	Survivors (*n* = 5,029)	Non-survivors (*n* = 191)	*P*-value
Age, years	52.6 ± 10.7	52.2 ± 10.6	62.7 ± 9.5	<0.001
Male sex, *n* (%)	2,461 (47.3)	2,337 (46.6)	124 (64.9)	<0.001
History of CVD, *n* (%)	379 (7.3)	337 (6.7)	42 (22.0)	<0.001
Current smoking, *n* (%)	1,356 (26.1)	1,277 (25.5)	79 (41.4)	<0.001
Diabetes, *n* (%)	326 (6.3)	282 (5.6)	44 (23.0)	<0.001
BMI (kg/m^2^)	25.6 ± 4.4	25.6 ± 4.4	26.7 ± 5.5	<0.001
Hypertension, *n* (%)	1,723 (33.1)	1,613 (32.2)	110 (57.6)	<0.001
SBP (mmHg)	127.6 ± 17.7	127.2 ± 17.6	135.3 ± 19.1	<0.001
eGFR (ml/min/1.73 m^2^)	78.7 ± 15.7	78.8 ± 15.5	75.4 ± 20.0	0.014
Total cholesterol (mmol/l)	5.56 ± 1.02	5.57 ± 1.01	5.53 ± 1.19	0.625
HDL cholesterol (mmol/l)	1.64 ± 0.44	1.64 ± 0.44	1.54 ± 0.47	0.001
LDL cholesterol (mmol/l)	3.32 ± 0.91	3.32 ± 0.90	3.26 ± 1.02	0.362
Triglycerides (mmol/l)	1.37 ± 1.14	1.36 ± 1.06	1.78 ± 2.33	<0.001
SCORE risk (%)	2.08 ± 3.57	1.91 ± 3.35	6.01 ± 5.80	<0.001
Known AD (RA, SLE)	115 (2.2)	110 (2.2)	5 (2.6)	0.696
Anti-apoA-1 IgG positivity	1,035 (19.9)	984 (19.6)	51 (26.7)	0.016
Anti-apoA-1 IgG levels (AU)	0.39 [0.34]	0.39 [0.33]	0.43 [0.40]	0.007
Incident overall death rate, *n* (%)	191 (3.7)		191 (3.7)	
Cancer-related, *n* (%)	69 (36.1)		69 (36.1)	
CVD-related, *n* (%)	36 (18.9)		36 (18.9)	
Infectious-related, *n* (%)	25 (13.1)		25 (13.1)	
Other causes^a^, *n* (%)	51 (26.7)		51 (26.7)	
Undetermined, *n* (%)	10 (5.2)		10 (5.2)	

*P values are derived from the comparison of patients with low vs. high levels of anti-apoA-1 IgG. Variables are expressed as mean ± SD or median [interquartile range] as appropriate or number of participants and percentage. Statistical analysis for continuous variables was performed by Student’s *t*-test or Mann–Whitney test depending on the normality assumption. Chi-squared test was used for categorical variables*.

*AD, autoimmune disease; CVD, cardiovascular disease; SBP, systolic blood pressure; eGFR, estimated glomerular filtration rate according to the MDRD formula; HDL, high-density lipoprotein; LDL, low-density lipoprotein; RA, rheumatoid arthritis; SLE, systemic lupus erythematosus; SCORE, Systematic Coronary Risk Evaluation; AU, arbitrary units of optical density; BMI, body mass index; anti-apoA-1 IgG, autoantibodies against apolipoprotein A-1*.

*^a^Other causes include lung disease (*n* = 12), bleeding and trauma-related complications (*n* = 11), suicide (*n* = 9), chronic renal failure (*n* = 6), non-ischemic heart failure (*n* = 5), chronic liver failure (*n* = 5), and dementia (*n* = 3)*.

Kaplan–Meier curves for participants with positive and negative anti-apoA-1 IgG titers are shown in Figure 1B; participants positive for anti-apoA-1 IgG had higher mortality rates than participants negative for the marker (4.9 vs. 3.4%, log-rank *P* = 0.034). Conversely, 95.1% of anti-apoA-1 IgG-positive subjects survived, compared to 96.6% of anti-apoA-1 IgG-negative subjects corresponding to an absolute survival difference of 1.5% between the two groups.

Cox regression analysis indicated that anti-apoA-1 IgG positivity was associated with a 1.5-fold increased risk of death, and this hazard rate remained unchanged after adjustment for the aforementioned mortality risk factors, including baseline CVD and AD: HR = 1.54 (1.11–2.13), *P* = 0.01. Similarly, 1 SD increase of the log-transformed anti-apoA-1 IgG levels was associated with a 15% increase in the risk of all-cause mortality: HR = 1.15 (1.02–1.28), *P* = 0.028 (Table 2). Sensitivity analyses excluding participants with CVD or AD at baseline led to comparable findings (Table 2).

**Table 2:** **Association between anti-apoA-1 IgG and all-cause mortality**.

	Events (*n*)	Unadjusted HR HR (95% CI)	*P*-value	Adjusted HR^a^ HR (95% CI)	*P*-value
Overall sample (*n* = 5,220)					
– High vs. low levels – For 1 SD increase in log (anti-apoA-1 IgG)	191 191	1.41 (1.02–1.95) 1.14 (1.01–1.28)	0.035 0.040	1.54 (1.11–2.13) 1.15 (1.02–1.30)	0.010 0.028

Participants without CVD at baseline (*n* = 4,825)					
– High vs. low levels – For 1 SD increase in log (anti-apoA-1 IgG)	149 149	1.42 (0.98–2.04) 1.14 (0.99–1.31)	0.062 0.062	1.62 (1.12–2.34) 1.19 (1.03–1.38)	0.011 0.019

Participants without AD at baseline (*n* = 5,105)					
– High vs. low levels – For 1 SD increase in log (anti-apoA-1 IgG)	186 186	1.42 (1.02–1.97) 1.15 (1.01–1.30)	0.036 0.030	1.68 (1.21–2.34) 1.23 (1.07–1.40)	0.002 0.002

*Data are expressed as adjusted HRs and 95% CI. Statistical analysis by Cox proportional hazards regression*.

*AD, autoimmune disease; HR, hazard ratio; CVD, cardiovascular disease; 95% CI, 95% confidence interval; anti-apoA-1 IgG, autoantibodies against apolipoprotein A-1*.

*^a^Adjusted for age, sex, hypertension, diabetes, smoking, high- and low-density lipoprotein cholesterol, body mass index, estimated glomerular filtration rate baseline CVD and AD*.

**Figure 2:**
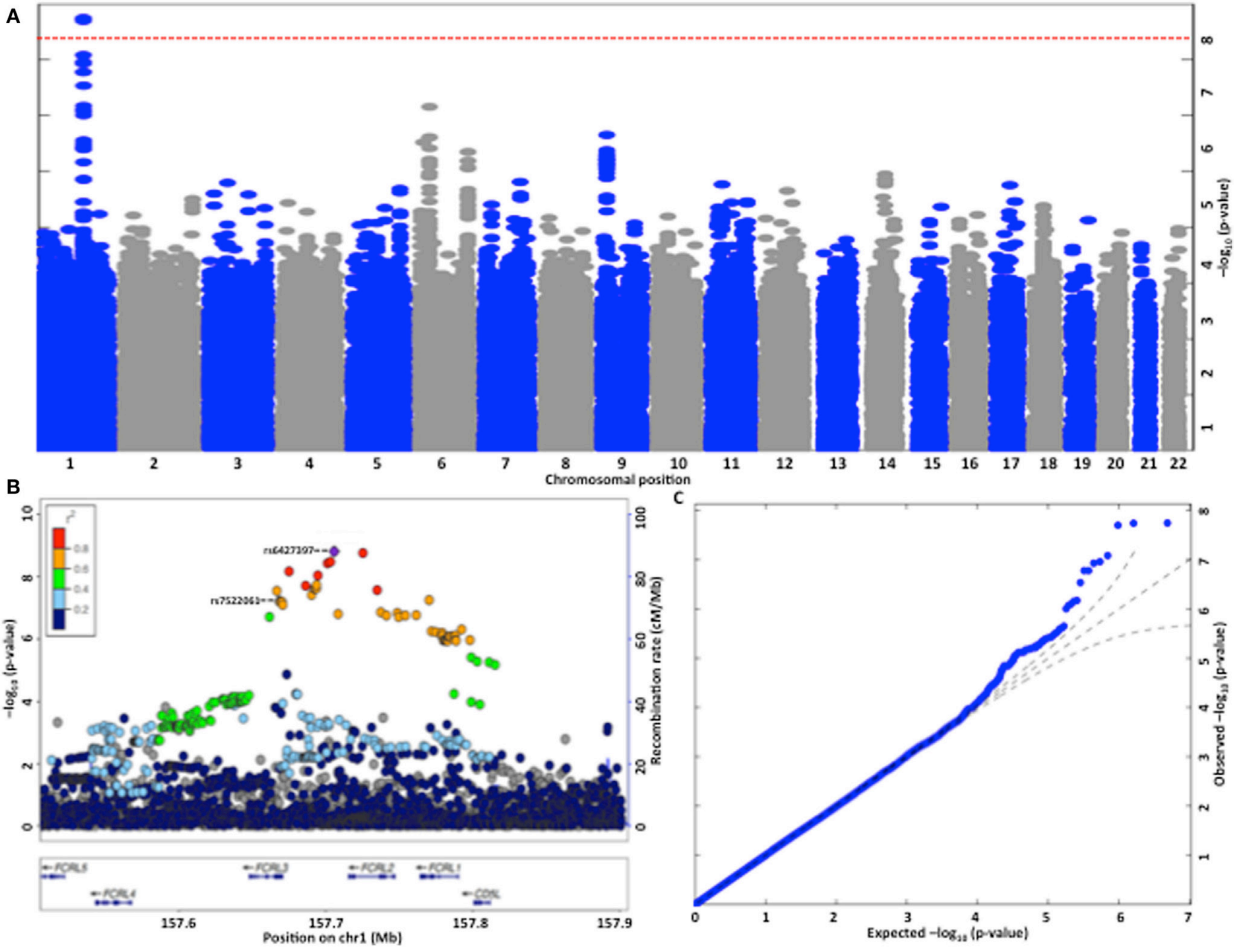
**(A)** Manhattan plot of the genome-wide association analysis for autoantibodies against apolipoprotein A-1 (anti-apoA-1 IgG) levels. The *x*-axis is chromosomal position and the *y*-axis is the significance (−log *P*; two-tailed) of association derived by the linear regression. The red line represents genome-wide significance level (*p* = 5 × 10^−8^) that was achieved by a single locus located in chromosome 1. **(B)** Regional plot at the Fc receptor-like (FCRL)2/3 locus on chromosome 1 showing single-nucleotide polymorphism (SNP) association strength for anti-apoA-1 IgG levels. To fine map the genome-wide significant association at the FCRL2/3 locus, we re-imputed the 400-kb window around the top HapMap-associated SNP using haplotypes from the HRC reference panel. −log10 *P* values (*y*-axis) are presented according to their chromosomal positions (*x*-axis). The index SNP in the analysis (i.e., SNP with the smallest *P*-value, rs6427397, purple diamond) and the only coding SNP identified (rs7522061) are labeled. The color intensity of each symbol reflects their linkage disequilibrium (*r*^2^) values with the index SNP, red (*r*^2^ > 0.8) through to navy blue (*r*^2^ < 0.2). Genes in each region, direction of transcription, and genomic coordinates are shown at the bottom. **(C)** Quantile–quantile plot of SNPs for anti-apoA-1 IgG levels. The negative logarithm of the observed (*y*-axis) and the expected (*x*-axis) *P*-value is plotted for each SNP (dot). The black lines indicate the null hypothesis of no true association (*x* = *y*) with 95% confidence intervals. Significant deviation from the expected (null) *P*-value distribution is evident only in the upper tail area (blue line), corresponding to SNPs with the strongest association.

Genome-wide association study on anti-apoA-1 IgG levels identified a single locus located in chromosome 1 (157.6–157.9 Mb) including 21 SNPs significantly associated with anti-apoA-1 IgG levels (Figure 2A; Table S2 in Supplementary Material). The T allele of the lead SNP (rs6427397) was associated with an increase of anti-apoA-1 IgG levels by 0.113 AU and explained 0.67% of anti-apoA-1 IgG variation. Conversely, in logistic regression (yes vs. no anti-apoA-1 IgG positivity) analysis, the T allele of rs6427397 had an odds ratio (OR) of 1.27 (95% CI 1.15–1.40) for anti-apoA-1 IgG positivity in our sample. The 20 remaining SNPs identified in the same region were in strong linkage disequilibrium and associated with anti-apoA-1 levels at *P*-value <10^−7^ (Figure 2B). Most of these SNPs were located in or near the Fc receptor-like 2 (*FCRL2*) and 3 (*FCRL3*) genes and to some extend in the regions spanning the *FCRL1* gene (Figures 2B,C). SNPs with a previously known biological function or association with a clinical trait were located in the *FCRL3* gene (Table S2 in Supplementary Material). Additionally, the only coding polymorphism among the identified SNPs represents a missense variant (rs7522061) also located in the *FCRL3* gene. We also observed an association between anti-apoA-1 IgG and a missense variant (rs1047989) in the human leukocyte antigen (HLA)-DQA1 gene, which did not, however, achieve stringent genome-wide significance (*P* = 7.27 × 10^−7^, data not shown).

## Discussion

This is the first study to demonstrate that anti-apoA-1 IgG levels are significantly and independently associated with all-cause mortality in the general population. In our population, after 5 years of FU, 95.1% of anti-apoA-1-positive subjects survived, compared to 96.6% of anti-apoA-1-negative participants. This corresponds to a modest, nevertheless, significant (*P* = 0.03) absolute survival difference of 1.5% between the two groups.

Our results extend previous findings (19, 22, 25, 26) and suggest that preclinical autoimmunity against apoA-1/HDL—which affects up to one-fifth of the general population—may identify individuals at increased risk of death. Our findings raise the possibility that presence of anti-apoA-1 IgG leads to pathophysiological events affecting not only CV prognosis, but also survival in the long term. Based upon previous studies, such events could be related to a chronic low-grade inflammatory state through sustained activation of the TLR2/TLR4/CD14 complex and production of pro-inflammatory cytokines (14, 18, 21, 24, 38), associations with elevated high-sensitivity C-reactive protein and uric acid levels (23, 25), impairment of HDL function (10–13, 16, 17), interference with basal heart rate regulation (22, 24, 25) or B-cell epitope spreading (39). Other pathophysiological mechanisms, other than those currently ascribed to anti-apoA-1 IgG could also be involved, in the same way that multiple molecular pathways underlie the associations of other autoantibodies with all-cause mortality in the community (7, 9, 40, 41). These challenging points will have to be investigated in future clinical and translational research efforts.

The second notable finding of our study is that anti-apoA-1 IgG levels are related to genetic polymorphisms belonging or regulating the *FCRL3* gene. Indeed, our lead SNP (rs6427397) is an intergenic variant that represents a strong expression quantitative trait loci for the *FCRL3* gene in whole blood (1.2 × 10^−12^) (42). Additionally, among the 20 remaining *FCRL* SNPs achieving genome-wide significance, the only coding variant identified (rs7522061) is a missense variant of the *FCRL3* gene.

Fc receptor-like genes are located in the human chromosome regions 1q21–23 and belong to the immunoglobulin genes superfamily. *FCRL3* in particular is known to encode a mature B-cells co-receptor primarily expressed in secondary lymphoid organs and involved in B cell maturation, regulation, and production of autoantibodies (43–45). Previous work suggests that *FCRL3* expression further affects T regulatory cells development and function, with high expression resulting in abnormal immune activation and breakdown of self-tolerance (46). Corroborating these *in vivo* findings, large-scale GWAS have identified *FCRL3*-related SNPs as major susceptibility genes for numerous ADs in humans (44, 47, 48).

The observed association of *FRCL3* with anti-apoA-1 IgG values in our sample is in line with previous studies showing that two-thirds of candidate loci for autoimmunity discovered by GWAS represent shared risk factors for multiple ADs (1, 3, 49). Among potential pathophysiological mechanisms, *FCRL3* may predispose to clinical autoimmunity by pleiotropic regulation of the production of other deleterious autoantibodies. *FCRL3* has been associated with the production of cyclic citrullinated peptide autoantibodies in rheumatoid arthritis (RA) (47) and antibodies to thyroid peroxidase in patients suffering from Graves’ disease (33). In a study of genetic determinants of autoantibody production in over 8,000 type 1 diabetes cases, Plagnol et al. (31) identified the *FCRL3* locus to be associated with antibodies against insulinoma-associated antigen 2 concluding that *FCRL3* “may have general effects in adaptive immunity, in the complex interactions between antigen presenting cells and T cells leading to antibody producing plasma B cells.” Unfortunately, we were unable to measure in our sample other antibodies, such as anti-oxidized LDL, antiphospholipid, antinuclear, or anti-heat shock protein antibodies in order to test this hypothesis.

Finally, with regards to anti-apoA-1 IgG and CV disease and mortality (19, 20, 22), *FCRL3* mRNA levels have been reported to be downregulated in patients with myocardial infarction when compared to those with stable angina or healthy subjects (50), suggesting that *FCRL3*-mediated immune dysregulation may also be involved in atheromatous plaque instability and rupture.

Although statistically significant, the effect of the T allele of the lead SNP (rs6427397) on autoantibody levels was modest [OR 1.27 (95% CI 1.15–1.40)] and explained 0.67% of total anti-apoA-1 IgG variation. This is not surprising since incremental effect sizes of genetic variants are rather common in genetic studies of autoimmunity where most risk alleles have ORs less than 1.2 (29, 30, 32) in spite of their low performance regarding disease prediction, these risk variants may provide important etiological information based on associated genomic regions, that could lead to a more sophisticated understanding of the molecular pathways underlying common ADs. Thus, genetic signatures of susceptibility to autoimmunity could provide a basis for assessing heterogeneity in disease progression, in response to targeted immune-modulating interventions, as well as for rational novel drug design.

The fact that the lead SNP in our study relates to an intergenic non-coding variant in the *FCRL3* locus is in accordance with current evidence from high-density genotyping and epigenomic studies which demonstrate that in common ADs, up to 90% of identified causal variants appear to be non-coding, while 60% correspond to immune cell enhancers (3). The current paradigm supports the notion that intergenic regions are densely populated with hundreds of thousands of regulatory elements that modulate cell type-specific gene expression (1, 3). In a study on RA (47), Kochi et al. demonstrated that non-coding SNPs in the promoter region of *FCRL3* have a regulatory effect on expression of the *FCRL3* gene and relate to augmented autoantibody production in subjects with the susceptible genotype. In the same line of thought, our present results indicate that the sequence of events leading to high of anti-apoA-1 IgG levels could be related to an impaired regulation of gene expression programs—including *FCRL3*—a hypothesis that requires further study.

Finally, although we did not retrieve any strictly genome-wide significant association between anti-apoA-1 IgG and HLA-related genes, we did observe an association trend at *P* = 7.27 × 10^−7^ that may represent a clinically meaningful signal in future studies and further relates the presence of these autoantibodies to autoimmunity susceptibility genes.

Study limitations are worth noting. First, our genetic analysis was conducted in a single population sample, including only individuals of Caucasian ancestry and will require further validation in independent cohorts, ideally involving other ethnic groups. Since genetic data on anti-apoA-1 IgG are so far inexistent, we were unable to identify a replication cohort for our GWAS. Nevertheless, our findings represent the first attempt to identify the genetic determinants of anti-apoA-1 IgG. Second, as the specific functionality of T allele of rs6427397 is currently unknown, we can only so far extrapolate on the pathophysiological relevance of the reported association, as related to regulation of the *FCRL3* gene. Indeed, the only genome-wide significant coding variant identified (rs7522061) is a missense variant of the *FCRL3* gene. As we only measured baseline anti-apoA-1 IgG levels, we were not able to assess the dynamic of anti-apoA-1 IgG levels over time in relation with mortality. Knowing whether an increase of anti-apoA-1 IgG would precede clinical events or whether our findings could be affected by transient anti-apoA-1 IgG positivity in a single sample will be important to determine in future studies.

Similarly, one could argue that since the actual difference in anti-apoA-1 IgG levels between the survivors and non-survivors was modest, these results are unlikely to be clinically relevant even though the relative observed difference in anti-apoA-1 IgG values is 9.3% and statistically significant in our sample (*P* = 0.007), as expected from sample power calculation. The fact that the distribution of anti-apoA-1 IgG values is positively skewed (25) and that low CV risk participants are known to present lower levels of anti-apoA-1 IgG than high CV risk subjects (19, 22, 25) could explain these relatively low anti-apoA-1 IgG values in the general population, despite significant differences between survivors and non-survivors. Finally, the observed anti-apoA-1 IgG values translates in a 19.9% anti-apoA-1 IgG positivity in the sample, which is similar to the reported prevalence of other IgG autoantibodies in the community (7).

Despite these limitations, our study provides—to our best knowledge—the first evidence of a significant association of anti-apoA-1 IgG with all-cause mortality and a major AD susceptibility gene. Although our findings are in accordance and extending previous results, they will need to be replicated in independent, adequately powered, prospective cohorts before any conclusion on potential clinical implications can be drawn.

## Conclusion

Our study suggests that anti-apoA-1 IgG levels predict all-cause mortality in the general population and are associated with a single locus involving *FCRL3*, a gene known to predispose for ADs. Our findings indicate that preclinical autoimmunity to apoA-1 may identify a substantial proportion of individuals at increased risk of death in the general population, a finding that will need further validation in independent, prospective cohorts.

## Ethics Statement

The study was approved by the Institutional Ethics Committee of the University of Lausanne, and written informed consent was obtained from all participants before inclusion in the study, in accordance with the Declaration of Helsinki.

## Author Contributions

PA and PM-V contributed in study concept and design, analysis, and interpretation of data, statistical analysis and drafting of the manuscript, and critical revision of the manuscript for important intellectual content. JV, SP, NS, OH, and FM contributed in study concept and design, acquisition of the data, analysis and interpretation of the data, statistical analysis, and critical revision of the manuscript for important intellectual content. FM, ZK, GW, PV, and NV had study supervision, contributed in study concept and design, acquisition of data, analysis and interpretation of data, critical revision of the manuscript for important intellectual content, obtained funding, and provided study supervision, administrative, and technical support. All listed authors gave final approval of the manuscript to be published and agreed to be accountable for all aspects of the work, ensuring that questions related to the accuracy or integrity of any part of the work are appropriately investigated and resolved.

## Conflict of Interest Statement

The authors declare that the research was conducted in the absence of any commercial or financial relationships that could be construed as a potential conflict of interest.
