# Supplementary material for: Anti-Apolipoprotein A-1 IgG Predict All-Cause Mortality and Are Associated with Fc Receptor-Like 3 Polymorphisms
Source: Front Immunol. 2017 Apr 18;8:437. doi: 10.3389/fimmu.2017.00437 (PMC5394854; doi:10.3389/fimmu.2017.00437)
Supplement: Supplementary file 1 [file Data_Sheet_1.DOCX]

**Supplement**

**Anti-apolipoprotein A-1 IgG predict all-cause mortality and are associated with *FCRL3* polymorphisms**

**Supplementary table 1**: **Main characteristics of subjects included in the analysis *vs.* subjects missing follow-up data.**

| Overall sample  (n=6676) | Subjects included in the analysis  (n=5220) | Subjects missing follow-up data  (n=1456) | p-value |
| --- | --- | --- | --- |
| Age, years | 52.58 ±10.71 | 52.59 ± 10.84 | 0.979 |
| Male sex, n (%) | 2496 (47.30) | 693 (47.60) | 0.841 |
| History of CVD, n (%) | 398 (7.54) | 90 (6.18) | 0.076 |
| Current smoking, n (%) | 1382 (26.19) | 430 (29.53) | 0.011 |
| Diabetes, n (%) | 336 (6.37) | 100 (6.87) | 0.492 |
| Hypertension, n (%) | 1756 (33.28) | 579 (39.77) | <0.001 |
| SBP (mm Hg) | 127.56 ± 17.70 | 129.43 ± 18.29 | <0.001 |
| Body mass index (kg/m^2^) | 25.65 ± 4.41 | 26.34 ± 4.85 | <0.001 |
| Total cholesterol (mmol/l) | 5.56 ± 1.02 | 5.62 ± 1.10 | 0.093 |
| HDL cholesterol (mmol/l) | 1.64 ± 0.44 | 1.60 ± 0.43 | 0.006 |
| LDL cholesterol (mmol/l) | 3.32 ± 0.90 | 3.37 ± 0.95 | 0.038 |
| Triglycerides (mmol/l) | 1.37 ± 1.14 | 1.46 ± 1.31 | 0.010 |
| SCORE risk (%) | 2.07 ± 3.56 | 2.13 ± 3.40 | 0.145 |
| Anti-apoA-1 IgG, n (%) | 1040 (19.92) | 283 (19.80) | 0.920 |
| Anti-apoA-1 OD, AU | 0.39 [0.34] | 0.39 [0.35] | 0.377 |

Data are expressed as mean ± standard deviation or median [interquartile range] as appropriate or number of participants and (percentage). CVD; cardiovascular disease, SBP; systolic blood pressure, HDL; high density lipoprotein, LDL; low density lipoprotein, SCORE; Systematic Coronary Risk Evaluation, Anti-apoA-1 IgG, Autoantibodies against Apolipoprotein A-1, OD; Optical Density, AU; Arbitrary Units. Statistical analysis for continuous variables was performed using student’s t-test or Mann-Whitney test depending on the normality assumption. Chi-squared test was used for categorical variables.

**Supplementary Table 2: Summary of the most significant allelic associations (P value<5*10^-8^) with anti-apoA-1 IgG positivity**

| *SNP* | *chr* | *Position*  *Base pair* | *aA* | *aB* | *beta* | *SE* | *P* | *Gene* | *Function* | *Reported associations* |
| --- | --- | --- | --- | --- | --- | --- | --- | --- | --- | --- |
| rs6681271  *rs7522061  rs2210912  rs2210913  rs3761959  rs945635  rs7528684  rs2317230  rs2317231  rs6427394  rs2873405  rs2210918  rs1970265  rs1977710  rs11264804  rs1537949  rs10430455  *rs6427397  rs7529060  rs6675393  rs2050568 | 1  1  1  1  1  1  1  1  1  1  1  1  1  1  1  1  1  1  1  1  1 | 157666644  157668390  157668701  157668993  157669278  157670290  157670816  157674997  157686337  157690415  157692044  157692985  157693714  157693722  157694612  157701249  157703238  157705725  157725264  157734907  157770241 | T  T  A  C  C  C  A  G  G  C  T  T  G  A  C  T  T  C  T  C  C | C  C  C  T  T  G  G  T  T  T  G  C  C  G  T  C  A  T  G  T  T | 0.1065  0.1041  0.1043  0.1047  0.1046  0.1047  0.1039  0.1129  0.1083  0.1049  0.1062  0.1061  0.1065  0.1073  0.1096  0.1134  -0.1137  0.1179  0.1167  0.1079  0.1038 | 0.0192  0.0192  0.0192  0.0194  0.0194  0.0194  0.0194  0.0195  0.0193  0.0191  0.0190  0.0190  0.0191  0.0191  0.0191  0.0192  0.0192  0.0195  0.0194  0.0194  0.0191 | 2.8322e-08  6.1795e-08  5.8874e-08  6.3108e-08  6.5200e-08  6.3154e-08  7.9658e-08  6.8105e-09  1.9130e-08  3.8011e-08  2.4168e-08  2.4384e-08  2.3306e-08  1.8337e-08  8.9987e-09  3.6906e-09  3.3615e-09  1.5402e-09  1.7560e-09  2.6697e-08  5.5972e-08 | FCRL3  FCRL3  FCRL3  FCRL3  FCRL3  FCRL3  FCRL3  FCRL3  Unknown  Unknown  Unknown  Unknown  Unknown  Unknown  Unknown  Unknown  Unknown  FCRL2-3  FCRL2  FCRL2  FCRL1 | Intron  Missense  Intron  Intron  Intron  UTR-5  Promoter  Promoter  Unknown  Unknown  Unknown  Unknown  Unknown  Unknown  Unknown  Unknown  Unknown  Unknown  Intron  Intron  Intron | SHL  MS  Unknown  Unknown  RA, SLE, T1D, GD  RA, SLE, T1D, GD  RA, SLE, influences FRCL3 expression on B cells  Unknown  Unknown  Unknown  Unknown  Unknown  Unknown  Unknown  Unknown  Unknown  Unknown  Unknown  Unknown  Unknown  MS |

SNP: single nucleotide polymorphism, Chr: chromosome; SHL: sudden hearing loss; RA: rheumatoid arthritis; SLE: systemic lupus erythematosus; MS: multiple sclerosis; T1D: type 1 diabetes, GD: grave’s disease.

*identifies the two lead SNPs. The choice was based either on the smallest P value (rs6427397), or on SNPs corresponding to coding variants (rs7522061).
